# Determinants of Autonomy in Patients with Bipolar Disorder: The Role of Neurocognition and Emotion Regulation

**DOI:** 10.1192/j.eurpsy.2026.11219

**Published:** 2026-07-31

**Authors:** M. A. Yaiche, B. Aifa, S. Ben Othmen, A. Maaroufi, H. Mami, A. Aissa

**Affiliations:** 1 Sfax Faculty Of Medicine, Sfax; 2 Mohamed Taher Maamouri Hospital, Nabeul; 3 Tunis Faculty Of Medicine, Tunis; 4 Psychiatry, Mohamed Taher Maamouri Hospital, Nabeul, Tunisia

## Abstract

**Introduction:**

In patients with bipolar disorder (BD), psychosocial functioning, particularly autonomy, often remains impaired despite clinical remission. Several factors may influence this domain, including clinical profile, neurocognitive performance, and emotion regulation abilities. However, few studies have specifically examined the determinants of autonomy in euthymic patients with bipolar disorder.

**Objectives:**

To assess the impact of neurocognition and emotion regulation on autonomy among euthymic patients with bipolar disorder.

**Methods:**

A cross-sectional descriptive and analytical study was conducted among patients diagnosed with BD type I, II, or unspecified, who had been in euthymia for at least six months.

Autonomy was assessed using the *Functioning Assessment Short Test* (FAST), with the autonomy sub-score used to classify patients as having preserved or impaired autonomy.

Cognitive functions were evaluated using a battery of neuropsychological tests assessing verbal learning and memory, attention, and executive functions.

Emotion regulation was assessed using the *Difficulties in Emotion Regulation Scale* (DERS) and the *Regulation of Emotion Questionnaire* (REQ).

Statistical analyses explored correlations and associations between autonomy, clinical variables, cognitive performance, and emotion regulation indices (significance level p < 0.05).

**Results:**

A total of 60 patients were included.

Autonomy impairment was identified in 58% of the sample.

Patients with preserved autonomy demonstrated better cognitive performance, particularly in verbal learning (p = 0.039), semantic memory (p = 0.021), visuospatial working memory (p = 0.007), and cognitive flexibility (p = 0.039).

They also showed superior attentional performance (p = 0.045).

Emotionally, autonomous patients had significantly lower DERS scores, reflecting better overall emotion regulation (p < 0.001), especially in emotional clarity (p < 0.001), emotional acceptance (p = 0.002), impulse control (p < 0.001), and access to regulation strategies (p = 0.001).

Additionally, the use of functional internal emotion regulation strategies (REQ) was significantly associated with preserved autonomy (p = 0.018).

**Conclusions:**

This study highlights that autonomy in euthymic patients with bipolar disorder is closely linked to cognitive abilities and emotion regulation. Early identification of cognitive and emotional difficulties may guide targeted remediation and psychoeducational interventions aimed at improving autonomy and, more broadly, psychosocial reintegration in bipolar disorder.

**Disclosure of Interest:**

None Declared

